# Correlation Between Upper Dental Arch Dimensions and Vertical Facial Height in Subjects with Skeletal Class I Occlusion: A Cephalometric Analysis

**DOI:** 10.4317/jced.63264

**Published:** 2025-10-17

**Authors:** Alah Dawood Al-Dawoody, Shehab Ahmed Hamad

**Affiliations:** 1 BDS, MSc, PhD, MFDRCSI, MFDSRCPS. Assistant Professor of Orthodontics, Department of Pedodontics, Orthodontics, and Preventive Dentistry, College of Dentistry, University of Mosul, Mosul, Iraq; 2 BDS, MSc, MOMSRCPS, MFDTRCSED, FIBMS, FFDRCSI, FDSRCPS, FDSRCSEng, FICD. Professor of Oral and Maxillofacial Surgery, Kurdistan Higher Council of Medical Specialties, Erbil, Kurdistan Region, Iraq

## Abstract

**Background:**

This study investigates the association between upper arch dimensions and vertical facial height in individuals with Class I skeletal occlusion. The aim was to identify correlations that may inform orthodontic treatment planning.

**Material and Methods:**

A retrospective analysis was performed on 85 participants (43 females, 42 males) aged 18-25 years with Class I skeletal occlusion (ANB angle: 1-4°). Upper arch dimensions-intercanine width (ICW), interpremolar width (IPW), intermolar width (IMW), and arch length (AL)-were measured using digital dental models. Vertical facial height was assessed via digital cephalometric radiographs, including anterior facial height (AFH), posterior facial height (PFH), lower anterior facial height (LAFH), and the facial height ratio (FHR). Statistical analysis involved Pearson correlation coefficients and multiple regression.

**Results:**

Significant positive correlations emerged between upper arch dimensions and vertical facial height. ICW correlated moderately with AFH (r = 0.61, p &lt; 0.001) and LAFH (r = 0.57, p &lt; 0.001). IPW showed a significant association with PFH (r = 0.70, p &lt; 0.001), while IMW showed the strongest association with AFH (r = 0.73, p &lt; 0.001). The connection between AL and FHR was moderate (r = 0.54, p&lt;0.01). Males showed consistently higher correlations than females.

**Conclusions:**

There is a strong relationship between vertical face height and upper arch dimensions in Class I skeletal occlusion. These results highlight the clinical significance of evaluating vertical facial structure when planning an arch expansion or contraction treatment. Its potential as a predictor of vertical face growth pattern is shown by its substantial association with IMW, which may help with clinical decision-making.

## Introduction

Dental arch size and it's correlation with facial form is a major concern in the practice of orthodontics and dentofacial orthopedics. The accurate knowledge of these relations is necessary in the orthodontic diagnosis, planning of treatment, and prediction of results of treatment. Class I occlusion is the ideal occlusion and is the desired skeletal pattern with a good anteroposterior relationship of maxilla with mandible, is generally standardized goal of orthodontic treatment (1 , 2). Facial height, especially anterior and posterior vertical facial dimensions, is an essential element in facial aesthetics and occlusal function (3 , 4). The vertical dimension of the face has an effect not only on its aesthetic value but also on the arrangement and orientation of the dental arches. Earlier studies have conveyed views that variations in vertical facial patterns of individuals may be associated with different forms of dental arches which may be important for treatment planning and results (5 , 6). The inter-canine, inter-premolar and inter-molar widths in the upper arch together with the arch length are significant values in orthodontic diagnosis and treatment planning (7). They provide very useful information regarding the space within the arch for tooth alignment as well as possibilities of expanding the arch and describe its shape. Much has been written about the stability of these dimensional measures post-orthodontics, particularly relating them to issues of relation between arch form and facial morphology (8 - 10). Several studies have examined the relationship between facial height and the dimensions of the dental arch across various types of malocclusions (11 , 12). Nonetheless, there is a lack of targeted research on patients with Class I skeletal occlusion, who comprise a large segment of the orthodontic demographic. Gaining insight into these correlations in Class I patients is particularly crucial, as it is considered the main goal in the treatment for other malocclusion varities (13). The development of digital technology has increased the accuracy and reliability of dental model measurements and cephalometric analysis. Digital dental models offer detailed three-dimensional information about arch dimensions, while digital cephalometric analysis provides precise measurements of facial heights and angles. A comprehensive analysis of the relationship between facial anatomy and dental arch features is made possible by the integration of these technologies (14 , 15). This study used digital cephalometric analysis and digital dental models to investigate the connection between vertical face height and upper arch dimensions in people with Class I skeletal occlusion. According to the null hypothesis, patients with Class I skeletal occlusion do not significantly correlate vertical face height measurements with upper arch dimensions.

## Material and Methods

**Figure 1:**
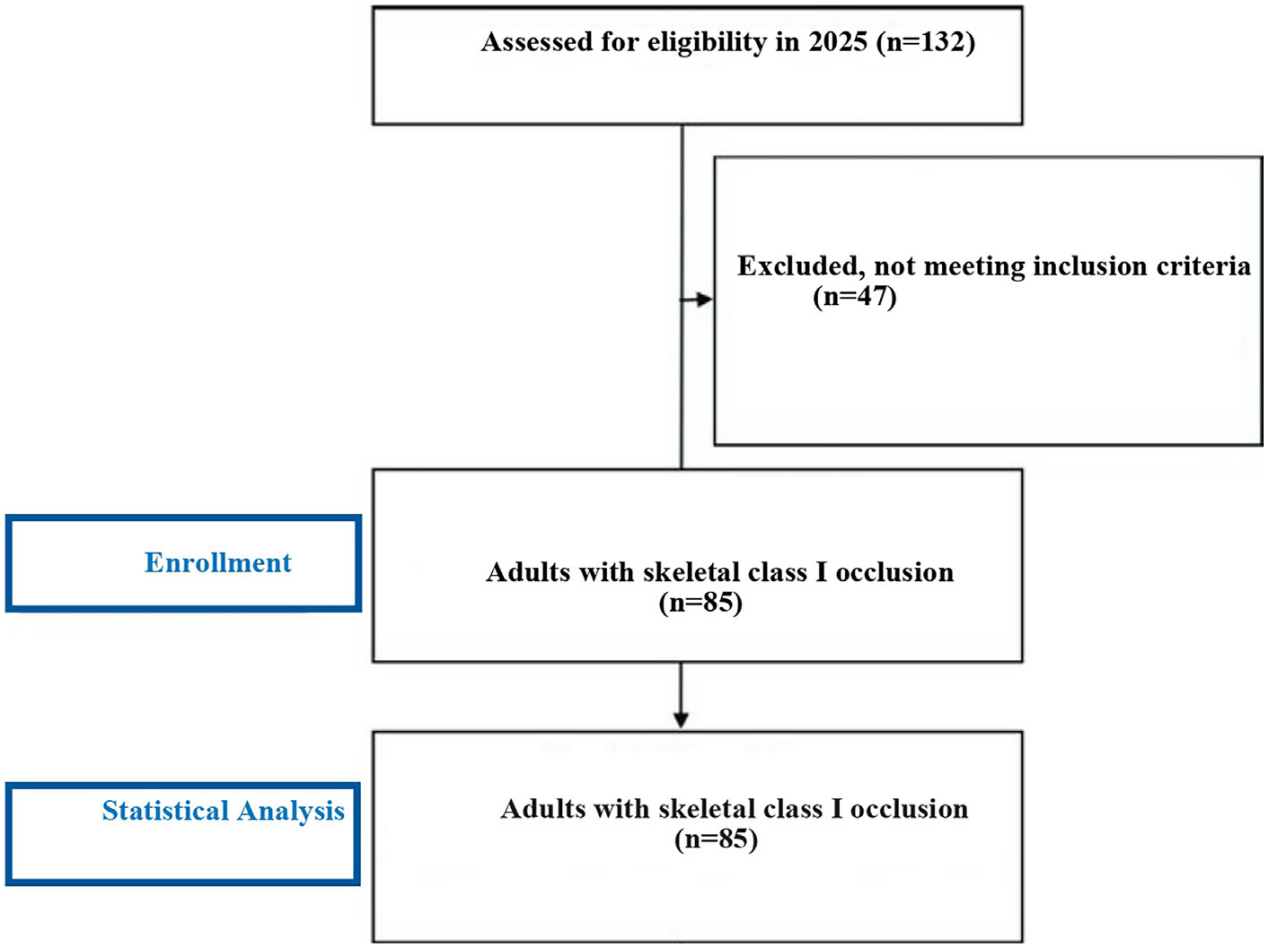
Flowchart of the study.

This retrospective cross-sectional study analyzed the pre-treatment records of patients requesting orthodontic treatment at a private dental clinic and private dental school in Erbil, Iraq, from July 2022 to May 2025 and institutional ethical approval was obtained. Figure 1 shows the flowchart of the study.

1

The inclusion criteria included skeletal class I occlusion (ANB angle 1-4°), age between 18-25 years, complete set of permanent teeth except third molars, no history of orthodontic treatment, orthognathic surgery, trauma, or facial deformity, and good quality cephalometric radiographs and digital dental models. The exclusion criteria included missing teeth (with the exception of third molars), extensive restorations, previous history of orthodontic treatment or orthognathic surgery, temporomandibular joint disorders, congenital craniofacial syndromes and developmental anomalies, and poor-quality radiographs or digital casts. We calculated sample size using G*Power 3.1.9.7 software with effect size 0.5, alpha error 0.05, and power 0.80. The minimum sample size was found to be 84 subjects. To account for potential dropouts and ensure adequate power, 85 subjects were included. Lateral cephalometric radiographs were analyzed using WEBCEPH software (version 1.0.3, Assemble Circle Co., Ltd., Seongnam-si, Gyeonggi-do, South Korea). All radiographs were taken with the same equipment under standardized conditions. The following vertical facial height measurements were recorded: 1. Anterior Facial Height (AFH): Distance from Nasion to Menton. 2. Posterior Facial Height (PFH): Distance from Sella to Gonion. 3. Lower Anterior Facial Height (LAFH): Distance from Anterior Nasal Spine to Menton. 4. Upper Anterior Facial Height (UAFH): Distance from Nasion to Anterior Nasal Spine. 5. Facial Height Ratio (FHR): LAFH/AFH × 100. Digital dental models were obtained using an intraoral scanner (Heyinscan S3, Loddon Medical Technology Co., Ltd., Shenzhen, China) and analyzed using 3Shape Ortho Analyzer software. The following upper arch dimensions were measured: 1. Intercanine Width (ICW): Distance between canine cusp tips. 2. Interpremolar Width (IPW): Distance between first premolar buccal cusp tips. 3. Intermolar Width (IMW): Distance between first molar mesiobuccal cusp tips. 4. Arch Length (AL): Distance from the contact point between central incisors to a line connecting the distal surfaces of first molars. Statistical analysis was conducted utilizing SPSS software (version 28.0, IBM Corporation, Armonk, NY). Descriptive statistics, including means, standard deviations, and ranges, were computed for all variables. The normal distribution was assessed using the Shapiro-Wilk test. Pearson correlation coefficients were determined to explore the relationships between upper arch dimensions and measurements of vertical facial height. Multiple regression analysis was carried out to uncover predictive relationships. Independent t-tests were utilized to compare measurements between male and female subjects. At p&lt;0.05, the threshold for statistical significance was set. Twenty randomly chosen participants were reassessed by the same examiner two weeks later in order to evaluate the measurement reliability. Intraclass correlation coefficients (ICC) were calculated to assess intra-examiner reliability. In order to assess inter-exa, 15 subjects were also measured by a different examiner.

## Results

The final sample consisted of 85 subjects (42 males, 43 females) with a mean age of 21.4 ± 2.2 years. The mean ANB angle was 2.3 ± 0.7°, confirming Class I skeletal occlusion in all subjects. Intra-examiner reliability showed excellent agreement with ICC values ranging from 0.91 to 0.97 for all measurements. Inter-examiner reliability demonstrated good to excellent agreement with ICC values ranging from 0.87 to 0.94, as shown in Table 1.

Table 1

The results, as presented in Table 2, demonstrate significant gender differences in upper arch dimensions.

Table 2

Males had larger inter-canine width (ICW: 35.0±1.9mm), inter-premolar width (IPW: 47.1±2.5mm), inter-molar width (IMW: 58.0±2.9mm), and arch length (AL: 39.3±2.3mm) compared to females (ICW: 33.2±1.8mm; IPW: 44.3±2.2mm; IMW: 54.6±2.7mm; AL: 37.7±2.1mm), with all differences being statistically significant (p0.001). Table 3 further shows that males also exhibited significantly greater vertical facial height measurements than females.

Table 3

These include anterior facial height (AFH: 122.1±6.1mm vs. 114.4±5.8mm), posterior facial height (PFH: 78.7±4.5mm vs. 73.5±4.1mm), lower anterior facial height (LAFH: 71.0±4.0mm vs. 66.2±3.7mm), and upper anterior facial height (UAFH: 51.1±2.9mm vs. 48.2±2.8mm), all with p-values &lt;0.001. No significant difference was found in facial height ratio (FHR) between genders (p=0.537). The correlation analysis of Table 4 revealed strong significant positive correlations between vertical facial height components and upper arch dimensions.

Table 4

IMW exhibited strongest correlations with AFH (r=0.73), PFH (r=0.65), LAFH (r=0.68), and UAFH (r=0.60), all significant at p&lt;0.001. IPW was similarly significantly correlated with PFH (r=0.70) and LAFH (r=0.62). ICW as well as AL revealed moderate to strong positive correlations with all vertical facial height parameters, particularly AFH and LAFH. Table 5 presents gender-specific correlation values with a more significant relationship in males than females.

Table 5

For example, for IMW-AFH, correlation was r=0.80 in males as opposed to r=0.64 in females, and for IPW-PFH, r=0.75 in males as opposed to r=0.63 in females. Corresponding trends were observed for ICW-LAFH and AL-FHR. These suggest that vertical facial height is more significantly related to dimensions of the upper arch in males than females.

## Discussion

The present study investigated the relationship between upper arch dimensions and vertical facial height in patients with Class I skeletal occlusion. The findings revealed significant positive correlations between all measured arch dimensions and vertical facial height parameters, rejecting the null hypothesis. These results provide valuable insights into the morphological relationships that exist in individuals with ideal skeletal patterns. The highest correlation was found between intermolar and anterior face height (r = 0.73), which means that persons with more vertical face height have more width in their buccal segments. This result is consistent with the theory that facial growth is a coordinated process, with vertical and transverse growth developing in a proportioned manner (16 , 17). This clinical connotation implies that patients with high anterior face height, may not need as much posterior transverse expansion, and that overexpansion of the arch, paradoxically, would also result in vertical openness, or broader arch because; on the other hand, patient who does not have high anterior face height, may need expansion farther back in the arch to develop the best arch form (18). The strong association between interpremolar width and posterior facial height (r = 0.70) indicates that the relationship of arch width to facial height is especially high in the posterior region. This strong association between interpremolar width and posterior face height is important in orthodontic treatment planning. Knowledge of this relationship may assist orthodontists in choosing treatment modalities, such as archwire selection and whether arch expansion is indicated. This relationship is particularly important for achieving facial aesthetics and harmony, as it influences the vertical dimension of the face. A study of Ocak et al. (1) demonstrated that the maxillary intermolar width was higher in hypodivergent-CI malocclusion (51.1 ± 3.4 mm) and significantly smaller in hypodivergent-CII-1 malocclusion (46.8 ± 3.4 mm). According to certain research, CII-1 malocclusion has a narrower maxillary posterior arch than Class I (CI) or ideal occlusion (19 , 20). Although the results were reported for female subjects, Foster et al. found no significant correlation between vertical morphology and maxillary intermolar width, which is in contrast to the results of the current study (2). Growth of the craniofacial region is influenced by the masticatory muscles. As the thickness of the masseter muscle increased, Tircoveluri et al. (21) and Biondi et al. (22) observed a decrease in vertical dimension and an increase in transverse growth. The observed gender differences in the strength of the correlations, wherein males consistently exhibit stronger correlations compared to females, may stem from growth differences between the two sexes. Males are known to have longer and later growth windows, which may result in stronger correlations between facial and dental structure (23). Gender-specific differences may be important when using these relationships in clinical settings because this data clearly highlights gender dimorphism, at least in some contexts. The current study's correlation levels are generally higher than those found in groups with mixed skeletal patterns, suggesting that Class I subjects may have stronger associations (24 , 25). This could indicate that the skeletal pattern of Class I bears a closer integration with the forms of the face and teeth. There are a number of reasons why the literature on arch widths is inconsistent, including gender dimorphism, racial and ethnic distortion, subject casting, sample size and selection, subject age, and measurement techniques. The findings of current study have many pertinent clinical implications for orthodontic treatment planning. First, the strong correlations imply that vertical facial features need to be taken into account when planning arch expansion or constriction type procedures. Longer-face patients may have a better tolerance for wider arches, while shorter-faced patients may have a better tolerance for narrower arches. Second, the predictive relationships identified through multiple regression analysis could be valuable for treatment planning. The ability to predict facial height from arch dimensions, or vice versa, could assist in establishing realistic treatment goals and expectations. Third, the stability of orthodontic treatment may be enhanced by respecting these natural morphological relationships. Treatments that maintain harmony between facial height and arch dimensions may be more stable in the long term. Several limitations should be acknowledged in this study. The cross-sectional design prevents assessment of developmental changes over time. A longitudinal study would provide more comprehensive understanding of how these relationships develop and change with growth. The study was limited to young adults aged 18-25 years, which may not represent relationships in other age groups. Additionally, the focus on Class I skeletal occlusion, while providing homogeneity, limits generalizability to other skeletal patterns. The measurements were limited to specific arch width dimensions and did not include arch depth or three-dimensional arch form analysis. Future studies incorporating comprehensive three-dimensional analysis may provide additional insights. Future research should investigate these relationships in other skeletal patterns to determine whether similar correlations exist in Class II and Class III individuals. Longitudinal studies following patients from childhood through adulthood would provide valuable information about the development of these relationships. Three-dimensional analysis incorporating arch depth and form could provide more comprehensive understanding of the relationship between facial morphology and arch characteristics. Additionally, investigation of how orthodontic treatment affects these relationships would be valuable for treatment planning.

## Conclusions

This study uncovers important relationships between upper arch measurements and vertical facial height in Class I skeletal individuals, with intermolar width being a strong predictor of anterior facial height and interpremolar width associated with posterior facial height. Males exhibit more significant correlations compared to females, emphasizing gender disparities. Clinically, these connections should inform orthodontic planning, particularly in arch widening or narrowing, to achieve the best results. Additional studies are required to investigate these relationships in different skeletal configurations and 3D elements for enhanced treatment accuracy and stability.

## Figures and Tables

**Table 1:** Sample Characteristics and Reliability Statistics.

Parameter	Value
Total Subjects	85
Males	42
Females	43
Mean Age	21.4 ± 2.2 years
Mean ANB Angle	2.3 ± 0.7° (Class I skeletal occlusion)
Intra-examiner ICC	0.91–0.97 (Excellent agreement)
Inter-examiner ICC	0.87–0.94 (Good to excellent agreement)

1

**Table 2:** Descriptive Statistics for Upper Arch Dimensions (mm).

Variable	Total Sample (n=85)	Males (n=42)	Females (n=43)	p-value
ICW	34.1 ± 2.0	35.0 ± 1.9	33.2 ± 1.8	<0.001*
IPW	45.7 ± 2.7	47.1 ± 2.5	44.3 ± 2.2	<0.001*
IMW	56.3 ± 3.1	58.0 ± 2.9	54.6 ± 2.7	<0.001*
AL	38.5 ± 2.4	39.3 ± 2.3	37.7 ± 2.1	0.001*

2

**Table 3:** Descriptive Statistics for Vertical Facial Height Measurements.

Variable	Total Sample (n=85)	Males (n=42)	Females (n=43)	p-value
AFH (mm)	118.2 ± 6.7	122.1 ± 6.1	114.4 ± 5.8	<0.001*
PFH (mm)	76.1 ± 4.8	78.7 ± 4.5	73.5 ± 4.1	<0.001*
LAFH (mm)	68.6 ± 4.2	71.0 ± 4.0	66.2 ± 3.7	<0.001*
UAFH (mm)	49.6 ± 3.1	51.1 ± 2.9	48.2 ± 2.8	<0.001*
FHR (%)	58.0 ± 2.7	58.1 ± 2.8	57.9 ± 2.6	0.537

3

**Table 4:** Pearson Correlation Coefficients Between Upper Arch Dimensions and Vertical Facial Heights.

Arch Dimension	AFH	PFH	LAFH	UAFH	FHR
ICW	0.61***	0.47**	0.57***	0.50**	0.41*
IPW	0.67***	0.70***	0.62***	0.55**	0.37*
IMW	0.73***	0.65***	0.68***	0.60***	0.44*
AL	0.58**	0.51**	0.53**	0.48**	0.54**

Significant: *p <0.05, **p <0.01, ***p <0.001.

**Table 5:** Gender-Specific Correlation Coefficients.

Variable Pair	Males (r)	Females (r)	Difference
IMW-AFH	0.80***	0.64***	0.16
IPW-PFH	0.75***	0.63**	0.12
ICW-LAFH	0.63***	0.50**	0.13
AL-FHR	0.60**	0.46*	0.14

Significant: *p <0.05, **p <0.01, ***p <0.001.

## Data Availability

The data available on request by contacting the corresponding author Shehab Ahmed Hamad.
